# *QuickStats:* Death Rates* from Colorectal Cancer,^†^ by Age Group — United States, 1999–2019

**DOI:** 10.15585/mmwr.mm7035a5

**Published:** 2021-09-03

**Figure:**
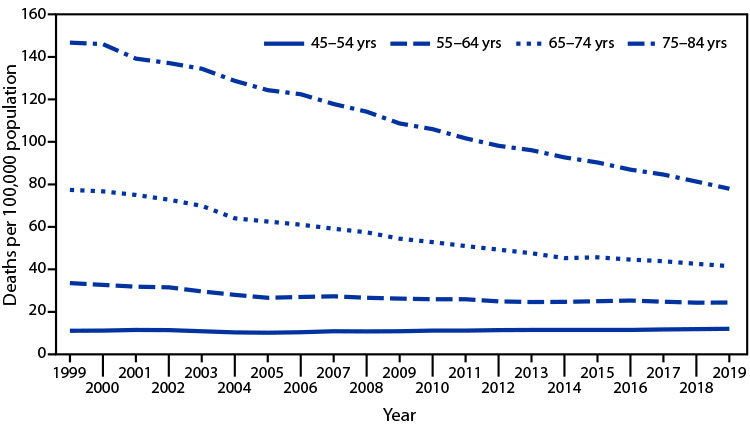
During 1999–2019, deaths per 100,000 persons from colorectal cancer decreased among persons aged 55–64 years (from 33.5 to 24.4), persons aged 65–74 years (from 77.4 to 41.5), and persons aged 75–84 years (from 146.7 to 77.9). The death rate from colorectal cancer among persons aged 45–54 years generally increased from 1999 (11.1) to 2019 (12.0). In each year during 1999–2019, the death rate was highest among persons aged 75–84 years and lowest among persons aged 45–54 years.

**Source:** National Center for Health Statistics, National Vital Statistics System, Mortality Data, 1999–2019. https://www.cdc.gov/nchs/nvss/deaths.htm

